# Preparation, physical properties, and evaluation of antioxidant capacity of aqueous grape extract loaded in chitosan‐TPP nanoparticles

**DOI:** 10.1002/fsn3.2891

**Published:** 2022-08-24

**Authors:** Sepideh Soleymanfallah, Zhaleh Khoshkhoo, Seyed Ebrahim Hosseini, Mohammad Hossein Azizi

**Affiliations:** ^1^ Department of Food Science and Technology North Tehran Branch Islamic Azad University Tehran Iran; ^2^ 68106 Department of Food Science and Technology Tehran Science and Research Branch Islamic Azad University Tehran Iran; ^3^ Department of Food Science and Technology College of Agriculture, Tarbiat Modares University Tehran Iran

**Keywords:** antioxidant, antioxidant activity, chitosan nanoparticles, food preservation, grape extract, ionic gelation

## Abstract

Grape extract is reportedly rich in phenolic compounds that possess strong antioxidant activities. Encapsulation of such extracts in nanoparticles (NPs) is an effective way to preserve various food products. In the present study, grapes were first extracted, and the amount of total phenolic content and different types of phenolic acids was determined. The extracts at different chitosan/extract weight ratios (1:0.25, 1:0.5, 1:0.75, and 1:1) were then encapsulated in chitosan nanoparticles (NPs) using the ionic gelation method. The extract‐loaded chitosan nanoparticles were characterized by their physicochemical properties using the dynamic light scattering (DLS) technique, chemical properties using Fourier‐transform infrared (FTIR) spectroscopy, and X‐ray powder diffraction technique (XRD), the morphological properties using scanning electron microscopy (SEM), and the antioxidant activity using the 2,2‐diphenyl‐1‐picrylhydrazyl (DPPH) test. The encapsulation efficiency (EE) and loading capacity (LC) were also assessed. Our findings showed that the free radical inhibition effect of NPs significantly increased with an increase in extract concentration. Chitosan NPs presented acceptable encapsulation efficiency and loading capacity (LC), and the encapsulation process enhanced the antioxidant activity of the free grape extracts. At the weight ratio of 1:0.5, the particle size and zeta potential of the NPs were 177.5 ± 2.12 nm and 32.95 ± 0.49 mV, respectively. FTIR and XRD analyses verified the credibility of the encapsulated grape extract in chitosan NPs. These NPs can be an efficient way to increase the shelf‐life of food products.

## Graphical abstract

The extracts at different chitosan/extract weight ratios were then encapsulated in chitosan nanoparticles (NPs) using the ionic gelation method. Then, the physicochemical and morphological properties, as well as antioxidant capacities, were investigated and characterized with the purpose to assess whether the shelf‐life would be prolonged via the use of chitosan nanocapsules.

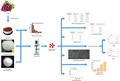

## 1 INTRODUCTION

For centuries, techniques in food research have evolved, and the use of conventional techniques has been systematically replaced by the use of complex mixtures and plant extracts (Zorzi et al., 2015). Grape extract is one of the richest sources of polyphenol compounds such as catechin, epicatechin, gallic acid, and pro anti cyanidin, as well as dimer, trimer, and tetramer compounds such as procyanidins. These compounds are also known for their antioxidant, antimicrobial, anticancer, and antiviral properties. Gallic acid in grape seed extract contains various flavonoids such as monomeric flavan–3‐ols such as catechin, epicatechin, gallocatechin, epigallocatechin, epicatechin 3‐O‐gallate, procyanidin dimers and trimers, and highly polymerized procyanidin. It has been reported that cardiac diseases, diabetes, and cancer may occur due to an antioxidant imbalance in the body, so the recommended intake of grape seed extract to prevent cardiovascular risk is between 100 and 300 mg/day (Gibis et al., 2013). However, including these nutraceutical compounds into food products in their pure form results in poor solubility and bioavailability, decreased shelf‐life, and activity loss during processing. Therefore, an effective delivery system should be engineered to overcome these disadvantages.

Since the beginning of the twentieth century, the food industry has exploited nanotechnology to synthesize food products to improve safety, prolong shelf‐life, and include the benefits of bioactive compounds. Nanoencapsulation offers several advantages over microencapsulation. The objectives of encapsulation are to preserve the material against environmental conditions such as light humidity and oxygen, increase the shelf‐life of products, and create a controllable condition of effective compounds. Such a nanoparticle (NP) system can encapsulate a more effective extract and generate higher thermodynamic stability. NPs have sizes ranging from 1 to a few hundred nanometers (nm). The selection of the nanoencapsulation technique usually depends on the nature of the bioactive compounds and encapsulating polymer. Selecting the suitable polymer will allow the synthesis of NPs with desired physicochemical properties, morphology, stability, and release kinetics (Čalija et al., 2017). To date, several encapsulating materials have been used to encapsulate bioactive compounds to manufacture functional foods. Among all these materials, chitosan has been widely investigated and considered a promising industry.

Chitosan is a widely used natural polysaccharide with antimicrobial and antioxidant activities (Omara et al., 2019). Owing to its nontoxic, biodegradable, and biocompatible nature, it is commonly used in different industrial sectors such as food, pharmaceuticals, agriculture, and environmental industries (Alves et al., 2018). Chitosan‐based materials are excellent candidates for the encapsulation of bioactive compounds to prolong the shelf‐life of industrially important food products. It has been proven that the encapsulation of herbal extracts such as grape seed extract with carriers like chitosan significantly improves the effectivity of the extract (Zarei et al., 2015). Compared to their normal form, chitosan NPs have a higher total volatile basic nitrogen (TVB‐N) inhibition effect. Ionic gelation is a more suitable method to prepare chitosan NPs to encapsulate bioactive compounds. The basis of ionic gelation relies on electrostatic interactions between the positively charged primary amino group of chitosan and negatively charged polyanion group like tripolyphosphate (TPP) (Chandrasekaran et al., 2020). This nontoxic process does not contain an organic solvent, and it is easy and controllable (Rasaee et al., 2016).

In the present study, we synthesized chitosan NPs containing grape extract at different concentrations for food preservation. The physicochemical, morphological, and chemical properties of the produced NPs were evaluated. The phenolic acid content and the total phenol content of the extract‐loaded chitosan NPs were determined. The characterized particles were then analyzed for their antioxidant properties. The chitosan/extract ratio of 1:0.5 was selected as the optimum value in our study due to the desired properties, encapsulation efficiency as well as loading capacity obtained. Our findings showed that grape extract‐loaded chitosan NPs are excellent candidates to prolong the shelf‐life of the food products and preserve foods from oxidation.

## 2 MATERIALS AND METHODS

### 2.1 Materials

Chitosan (acetylation‐grade 80%–85%, the molecular weight of 850 kD), penta‐sodium thymidine 5′‐triphosphate (TTP), and 2,2‐diphenyl‐1‐picrylhydrazyl (DPPH) were supplied by Sigma‐Aldrich (St. Louis, the USA). Acetic acid, sodium acetate, ethyl acetate, methanol (80%), dihydrogen phosphate, glycerol, acetonitrile, phenolic standards, Folin–Ciocalteu reagent (FCR), sodium carbonate, and gallic acid standard were procured from Merck (Germany). Ultrapure water was obtained using a purification device (FINETECH, FTWD‐501, Korea).

### 2.2 Extraction of grape

A previously published method was used with slight modifications (Rajaei et al., 2010). The grapes were first dried in an oven for 30 min at 50°C, and then the dried grapes were ground to obtain the powder, which was then passed through a mesh (No. 35). Afterwards, the obtained powder was dissolved in deionized water and placed in an ultrasonic bath (UP200H, Germany) at 55°C for 15 min exposed to 35 kHz waves. Then the extract was filtered using Whatman filter paper No. 5 to remove impurities. The obtained extract was then placed in a sealed container away from light and humidity.

### 2.3 Encapsulation of grape extract in chitosan NPs

Chitosan NPs were synthesized following the ionic gelation technique, which takes place through an electrostatic interaction between positively charged chitosan and negatively charged TPP (Dube et al., 2010; Ghaderi‐Ghahfarokhi et al., 2017; Ghaderi‐Ghahfarokhi et al., 2016; Haider et al., 2017; Zarei et al., 2015). Briefly, chitosan was dissolved in acetic acid (1% v/v), followed by sonification for 60 min to obtain a clear chitosan solution (2% w/v). The pH of the solution was stabilized between 3 and 4 using 4N NaOH solution. The TPP was dissolved in deionized water using a magnetic mixer at ambient temperature to obtain a TPP solution (2%). Afterwards, 4 ml of TPP was added to 100 ml of chitosan solution using a magnetic stirrer for 60 min at 700 rpm (revolutions per minute) to obtain a homogeneous solution. Different volumes of the extract in deionized water (0, 0.05, 0.75, 0.1, 0.125 v/v) were added to chitosan NPs and mixed with a magnetic mixer for 30 min to obtain different concentrations of chitosan‐to‐extract ratios (1:0, 1:0.25, 1:0.5, 1:075, and 1:1). After adding the extract, mixing was continued for an additional 30 min to achieve complete gelation.

### 2.4 Characterization of grape extract‐loaded chitosan NPs

#### 2.4.1 Physicochemical characterization

The physicochemical properties of the grape extract‐loaded chitosan NPs (particle size, polydispersity index (PDI), and zeta potential) were measured using the Dynamic Light Scattering (DLS) technique (Zetasizer Nano ZS, Malvern Instruments, the UK). The analyses were performed in triplicate in water at 25°C. The backscattering configuration was set with a detection angle of 90°. The helium–neon (He/Ne) laser emission and power source were 633 nm and 4.0 mW, respectively.

#### 2.4.2 Chemical characterization

The chemical properties of chitosan powder, grape extract, freeze‐dried chitosan NPs, and grape extract‐loaded chitosan NPs were measured using Fourier‐transform infrared spectroscopy (FTIR) (Nicolet IR 100, 4 cm resolution, 400–4000). The NP index was generalized and analyzed in X‐ray powder diffraction technique (XRD) (X’pert MPD, Philips, the Netherlands).

#### 2.4.3 Morphological characterization

To visualize the morphological properties of the NPs, Scanning Electron Microscopy (SEM) (JEOL, Japan, JSM 6400) was used. Images were developed for a chitosan/extract concentration ratio of 1:0.5.

#### 2.4.4 Encapsulation efficiency of chitosan NPs

The encapsulation efficiency of the chitosan NPs was determined according to the method previously described by Zhang et al. (2016), Tang et al. (2013), Shah et al. (2016) and Dube et al. (2010) with minor modifications. For quantitative examination of the amount of the extract loaded in chitosan NPs, they were first centrifuged for 1 h at 12,000 *g* at 4°C so that the sedimented NPs became floated. Glycerol was added to the solution to prevent particle coagulation. The amount of grape extract in the supernatant was determined by spectrophotometer at 250–400 nm (Cary 600, Agilent, the USA).

The HPLC was used to determine the concentration of the floated extract that represents the loading capacity of the chitosan NPs. Different concentrations of the extract (µg/ml) were prepared, and the adsorption level was read at 275 nm wavelength for each concentration using Perkin‐Elmer (series 600) equipped with a ultraviolet (UV) detector and reversed‐phase column (Pak C18, ODS1 4.5 mm * 150 mm). The injection volume was 10 ml with a flow discharge rate of 1.2 ml/min, system pressure of 10–15 Mpa, and operating temperature of 25°C. Before each injection, the device was calibrated, and the extract was measured at 280 nm wavelength.

The percentage of nanoencapsulation efficiency and loading capacity (LC) of the chitosan NP were obtained using Equations (1) and (2), respectively.

(1) Microencapsulation efficiency (%)=total encapsulated extract/total extract used×100

(2) $$\text{Loading capacity (\%)}=\left(\text{free extract}‐\text{total extract}\right)/\left(\text{free extract}\times \text{NPs weight}\right)*100$$

#### 2.4.5 Antioxidant activity

Antioxidant activity of the grape extract in free and encapsulated forms was measured following the method described by Tang et al. (2013) and Ghaderi‐Ghahfarokhi et al. (2017), Barzegar et al. (2016) with slight modifications. Different volumes of chitosan NPs containing the extract (1:0.5 weight ratio) were dissolved in ethanol (99%) to obtain the final concentration of 0.1–1 mg/ml. The samples were shaken at 200 rpm to separate the extract from chitosan NPs. Another solution with the same concentration (0.1–1 mg/ml) was prepared in ethanol, and then 0.3 ml of the extract (in a free and encapsulated form) at different concentrations and chitosan NPs were mixed. Then the mixtures were added into 2.7 ml of ethanol solution (6 × 10^−5^ mol/L) and then placed in a dark room for 30 min for the DPPH assay. Afterwards, the adsorption capacity of the mixture was measured at 517 nm against a blank (ethanol) using a UV spectrometer. To minimize the error coefficient, each measurement was repeated three times (Table 1). Free radical suppression percentage (DPPH) was obtained using Equation (3):

(3) $$\%\;\text{DPPH inhibition (I)}=\left(\text{A blank}‐\text{A sample}/\text{A blank}\right)*100$$

**TABLE 1:** The physicochemical properties of the grape extract‐loaded chitosan NPs at different chitosan/extract weight ratios

Chitosan/Extract (Weight ratio)	Particle size (nm)	PDI	Zeta potential (mV)
1:0	210 ± 1.41ᵈ	0.367 ± 0.008ᵃ	36.4 ± 0.14ᵉ
1:0.25	182.5 ± 2.12ᶜ	0.395 ± 0.004ᵇ	35 ± 0.28ᵈ
1:0.5	177.5 ± 2.12ᶜ	0.408 ± 0.003ᵇᶜ	32.95 ± 0.49ᶜ
1:0.75	139 ± 8.48ᵇ	0.437 ± 0.001ᵈ	26.45 ± 0.35ᵇ
1:1	118.5 ± 3.35ᵃ	0.426 ± 0.01ᶜᵈ	24.65 ± 0.21ᵃ

a, b, c and d: Diffrent letters in the same column indicates significant diffrences (*p* ≤ 0.05).

A blank is a light absorbed by the negative control without the extract, and A sample is light adsorbed with different concentrations of the extract.

#### 2.4.6 Phenolic acid content

The extraction and measurement of phenolic acid were carried out using the method previously described by Koponen et al. (2007) and Vekiari et al. (2008). To this end, 0.5 g of the extract was dissolved in deionized water, then mixed with ethyl acetate, and the final solution was passed through a filter (micro filter, CA‐45/25 S, Chromafil, Duren, Germany, 0.45 µm). Chromatographic separation was conducted in Hypersil ODS Column (4.6 × 250 ml) with a particle diameter of 5 µm at ambient temperature. Chromatography was determined using HPLC pump series crystal 200 (Unicom, the UK) equipped with ultraviolet–visible (UV–V) detector at 254 nm, corresponding to the maximum absorption value of the phenolic acid standard. The mobile phase used in the study included potassium dihydrogen phosphate and acetonitrile (20:0.8) with a flow rate of 1 ml/min. Phenolic acid standards included gallic acid, catechin, epicatechin, resveratrol, rutin, caffeic acid, ellagic acid, and quercetin. The standard solution of different phenolic acids was prepared at 1–400 mg/L of ethanol. The phenolic acids were examined with a 190–400 nm wavelength detector at 254 nm wavelength, and the results were expressed as µmol/wet weight (g).

#### 2.4.7 Phenol content assessment

Phenol content of the extract‐loaded chitosan NPs was assessed using the method previously described by Roostaee et al. (2017) and Zarei et al. (2015). Total phenol content was measured using the Folin–Ciocalteu assay. Briefly, 20 ml of the grape extract was mixed with 1.6 ml of distilled water and 100 ml of the Folin–Ciocalteu reagent (FCR). After 1 min, 300 ml of sodium carbonate solution (20%) was added. Test tubes were placed in a water bath at 40°C) after shaking, and after 30 min, the adsorption level was read at 760 nm using a spectrophotometer.

Standard gallic acid was used to create the standard curve. First, a gallic acid‐based solution was prepared at concentrations ranging between 10 and 100 mg/ml, and the standard curve was created based on the adsorption value against different concentrations of the acid. Total phenolic compounds in the extract were expressed as gallic acid in each gram of the extract. The measurements were carried out in triplicate.

#### 2.4.8 Statistical method

Data analyses were done in SPSS (ver. 19) and Excel 2020. Kolmogorov–Smirnov test was used to determine the normality of the data. Tukey test was used to compare the mean score of treatments. One‐way analysis of variance (ANOVA) (*p* < .05) was used to determine if the differences between each measured value were significant.

## 3 RESULTS AND DISCUSSION

### 3.1 Physicochemical properties

#### 3.1.1 Particle size and polydispersity index

The particle size (hydrodynamic diameter) of the NPs was measured using the DLS method. The diameter of the empty chitosan NPs was found to be 210 ± 1.41 nm (Table 1). Grape extract‐loaded chitosan NPs presented smaller diameters, and the size significantly decreased as the chitosan–grape extract mass ratio decreased. The particle size of the NPs with the ratios of 1:0.25, 1:0.5, 1:0.75, and 1:1 was 182.5 ± 2.12, 177.5 ± 2.12, 139 ± 8.48, and 118.5 ± 3.35 nm, respectively. No statistical significance (*p* > .05) was found between the ratios of 1:0.25 and 1:0.5. The difference in size between the samples of 1:0 and 1:0.25; 1:0.5 and 1:0.75, and 1:0.75 and 1:1 was found to be significant (*p* < .05). Previous studies have also reported that encapsulation of bioactive compounds such as plant extracts decreased the particle size (Ghaderi‐Ghahfarokhi et al., 2017; Ghaderi‐Ghahfarokhi et al., 2016; Haider et al., 2017; Roostaee et al., 2017; Yoksan et al., 2010).

In an aqueous medium, an increase in the size of extract‐loaded chitosan NPs can be less than empty NPs as the empty NPs can be hydrated as the extract with hydrophilic properties occupies the interior of chitosan NPs. Additionally, the degree of degradability of the chitosan NPs may vary during the encapsulation process, which results in the formation of particles of smaller size (Ghaderi‐Ghahfarokhi et al., 2017; Barzegar et al., 2016). Prior to measuring particle size using DLS, the samples were sonicated, and ultrasonic waves possibly broke the glycosidic bonds 1 and 4. Ultrasound cavitation destroys and decomposes long chitosan chains and converts them into smaller particles. Through this, the formation of bigger particles during ionic gelation is suppressed (Tang et al., 2013).

Polydispersity index (PDI) indicates the homogeneity of particles in a colloidal suspension (Cho et al., 2013). The particle size and PDI are affected by several factors, such as the molecular weight of the encapsulated compound, polymer material, and mixing method (Barzegar et al., 2016). As shown in Table 1, the decrease in the chitosan/extract ratio leads to the generation of polydisperse NPs. The PDI of empty chitosan NP is equal to 0.36 ± 0.008. Compared to empty NPs, the increase in PDI of the extract‐loaded NPs was statistically significant (*p* < .05). However, no significant difference was found between the ratios of 1:0.25 and 1:0.5 as well as 1:0.75 and 1:1. Encapsulation of plant extracts has been shown to increase the polydispersity of the NPs (Ghaderi‐Ghahfarokhi et al.,  2017; Barzegar et al., 2016; Haider et al., 2017; Roostaee et al., 2017) Ha et al. (2013).

#### 3.1.2 Zeta potential

Zeta potential indicates the surface charge of the particles, the degree of electrostatic repulsion, and their stability in a colloidal system. The particles with the zeta potential of less than −30 mV or greater than +30 mV are considered colloidally stable (Zarei et al., 2015). In our study, empty chitosan NPs had the zeta potential of 36.4 ± 0.14 mV. As shown in Table 1, the zeta potential of the particles decreased significantly as the grape extract was encapsulated in chitosan NPs.

The particles showed a declining trend as the chitosan/extract ratio decreased. The zeta potential values for the chitosan/extract ratios of 1:0.25, 1:0.5, 1:0.75, and 1:1 were 35 ± 0.2, 32.95 ± 0.5, 26.4 ± 0.3, and 24.6 ± 0.2 mV, significantly. Previous studies have also reported similar results (Ghaderi‐Ghahfarokhi et al., 2017; Barzegar et al., 2016; Haider et al., 2017; Keawchaoon & Yoksan, 2011). The decrease in the zeta potential of extract‐loaded chitosan NPs is expected as the plant extract containing phenolic compounds usually presents low zeta potential.

### 3.2 Encapsulation efficiency and loading capacity of chitosan NPs

Encapsulation efficiency is defined as the percentage of the grape extract entrapped in chitosan NPs. The efficiency strongly depends on the ratio between chitosan/grape extract. Moreover, the efficiency varies as the solubility of the extract in the matrix material or polymer changes (Kumari et al., 2010). In our study, the encapsulation efficiency was found to be between 51.90 ± 1.33 and 33.56 ± 0.41% (Table 2). The highest efficiency was obtained with the chitosan/extract ratio of 1:0.25. The efficiency significantly declined as we decreased the weight ratio between chitosan and grape extract. The main reason behind this declining trend is that the saturation level increases, and the excess grape extract cannot be adsorbed by chitosan NPs and can be easily separated during the centrifuge step of NP preparation. Keawchaoon and Yoksan (2011) previously reported similar results; Haider et al. (2017); Yoksan et al. (2010); Zhang et al. (2016); Ghaderi‐Ghahfarokhi et al. (2017); Barzegar et al., (2016) and Alishahi et al. (2011) and Ha et al. (2013). The molecular weight of the compound to be encapsulated significantly affects the encapsulation efficiency (Cai & Lapitsky, 2017). High‐molecular‐weight molecules usually have limited movement and lead to lower encapsulation efficiency. This is also highly related to the preparation method of the grape extract.

**TABLE 2:** Extract encapsulation efficiency (EE) and loading capacity (LC) of chitosan nanoparticles (NPs) at different chitosan/extract weight ratios

Chitosan/Extract (Weight ratio)	Encapsulation efficiency (%)	Loading capacity (%)
1:0	0	0
1:0.25	51.90 ± 1.33	4.2 ± 0.4
1:0.5	40.33 ± 0.8	5.78 ± 0.2
1:0.75	29.64 ± 1.36	6.31 ± 0.7
1:1	33.56 ± 0.41	6.7 ± 0.8

Loading capacity (LC) is the amount of the grape extract encapsulated per unit weight of the chitosan NP. It is calculated by the amount of total entrapped grape extract divided by the total chitosan NP weight. The loading capacity (LC) of the chitosan NPs ranged from 4.2 ± 0.4 to 6.7 ± 0.8% (Table 1). The lowest loading capacity was obtained at a chitosan/extract weight ratio of 1:0.25. As the ratio decreased, the loading capacity showed an increasing trend. However, after reaching the ratio of 1:0.75, the increase was insignificant. Previous studies have reported that an enormous concentration of the compounds (i.e., essential oils or plant extracts) leads to an increase in loading capacity (Ghaderi‐Ghahfarokhi et al., 2017; Barzegar et al., 2016; Haider et al., 2017; Tang et al., 2013; Keawchaoon & Yoksan, 2011; Yoksan et al., 2010;).

### 3.3 Determination of the antioxidant activity

**FIGURE 1:**
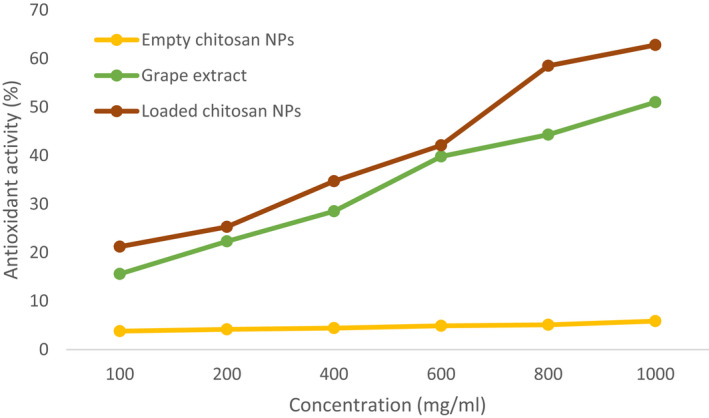
Antioxidant activity of the free grape extract (GE), empty chitosan NPs (Empty CS‐NP), and extract‐loaded chitosan NPs (Loaded CS‐NP) at the extract/chitosan weight ratio of 1:0.5

As shown in Figure 1, the antioxidant activity of the grape extract in free form ranged from 15.6% to 51.01%, whereas the encapsulated extract ranged from 21.2% to 62.8%. It can be observed that the increasing amount of encapsulated grape extract in chitosan NPs significantly enhanced the antioxidant activity (Figure 1). Additionally, a higher chitosan‐to‐extract ratio leads to a higher antioxidant activity. This result was also correlated with the encapsulation efficiency. There may be different reasons behind this finding. Phenolic compounds in the grape extract are highly volatile and can easily evaporate from the system. However, nanoencapsulation can preserve the phenolic compounds by decreasing the evaporation rate to suppress free radicals (Barzegar et al., 2016; Ghaderi‐Ghahfarokhi et al., 2017). As a control group, the antioxidant activity of the chitosan NPs was also evaluated. As shown in Figure 1, chitosan had an antioxidant activity of less than 10%. This is due to the hydroxyl and amino groups present in the chitosan backbone that can react with free radicals. During the ionic gelation process, amino groups of chitosan undergo cross‐linking with the polyanions present in TTP. Using different concentrations of chitosan leads to a varying level of cross‐linking, which results in an antioxidant activity ranging between 3.8% and 5.85%. Our findings were consistent with the previous investigations of Ghaderi‐Ghahfarokhi et al. (2017); Barzegar et al., (2016); Tang et al. (2013).

### 3.4 Phenolic acid profile and total phenol content of extract‐loaded chitosan NPs

**FIGURE 2:**
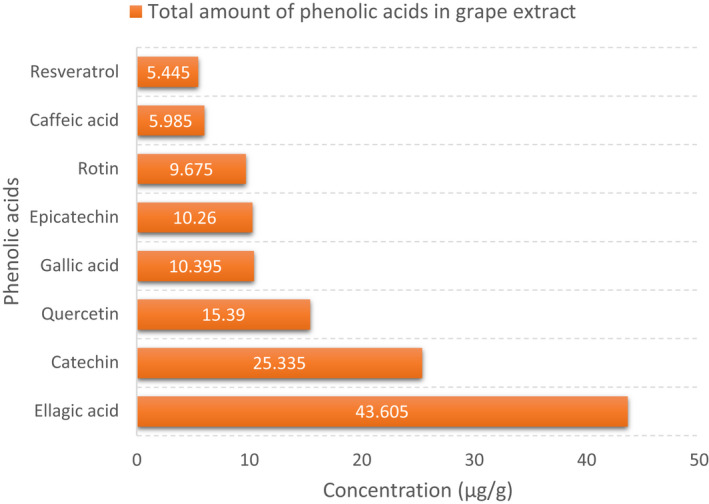
The concentration of phenolic acids (µg/g) in the grape extract (GE)

The grape was ground to obtain an extract, and the phenolic acid content of the extract was analyzed. As shown in Figure 2, the amounts of gallic acid, catechin, epicatechin, rutin, resveratrol, quercetin, ellagic acid, and caffeic acid were 10.395, 25.335, 10.26, 9.675, 5.445, 15.39, 43.605, and 5.985 µg/g, respectively. The highest and lowest phenolic acid contents in the grape extract were ellagic acid and resveratrol, respectively.

**FIGURE 3:**
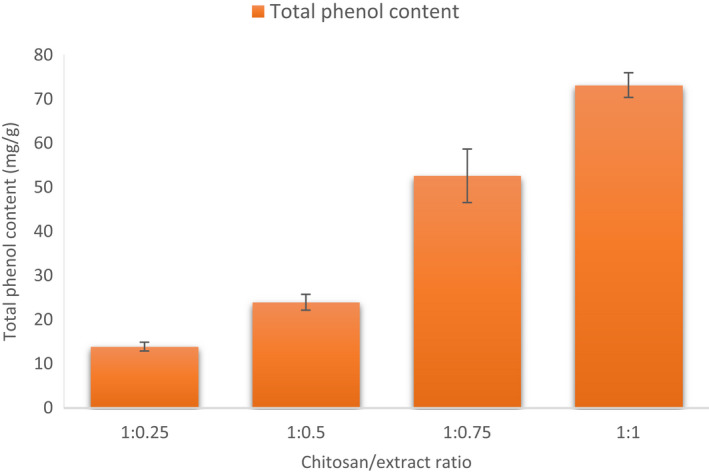
Total phenol content of extract‐loaded chitosan NPs (Loaded CS‐NP) at different chitosan/extract ratios

The total phenol content of the grape extract is demonstrated in Figure 3 for different chitosan/extract ratios (mg/g). The total phenol content significantly increased as the chitosan/extract ratio decreased (from 1:0.25 to 1:1). The highest total phenol content was obtained with a ratio of 1–1. The total phenol content of chitosan/extract ratios of 1:0.25, 1:0.5, 1:0.75, and 1:1 was determined to be 13.92 ± 1, 23.96 ± 1.8, 52.575 ± 6.04, and 73.095 ± 2.8 mg/g, respectively. The increase in phenol content between the ratios of 1:0.25 and 1:0.5 was not statistically significant (*p* > .05), while a significant difference was found between the ratios of 1:0.5 and 1:0.75, and between 1:0.75 and 1:1 (*p* < .05). The phenol content significantly varies depending on the plant extract. Roostaee et al. (2017) reported that the total phenol content of microencapsulated green pistachio‐loaded chitosan NP was 10 ± 0.05 mg/ml. Zarei et al. (2015) demonstrated that pomegranate and orange peel phenolic content in chitosan NPs was 364 ± 10.3 and 350 ± 6.4 mg TAE (tannic acid equivalent)/g. In a previous study, total phenol content in the grape peel extract and grape seed extract loaded with chitosan NPs was measured to be 42.064 ± 1.01 and 45.652 ± 3.74 mg/g, respectively (Silva et al., 2020).

### 3.5 Morphological characterization of NPs

**FIGURE 4:**
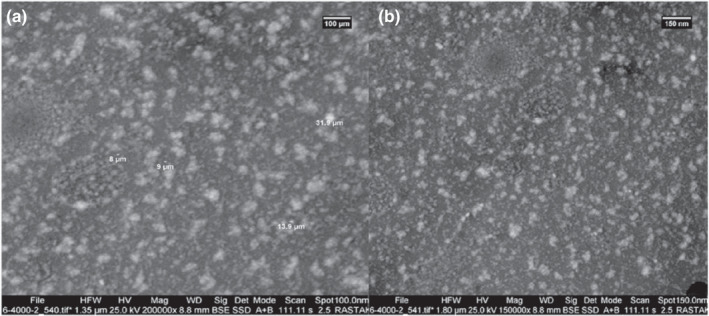
Scanning electron microscopy (SEM) images of empty (A) (Empty CS‐NP) and extract‐loaded chitosan NPs (Loaded CS‐NP) (B) at the chitosan/extract ratio of 1:0.5

**FIGURE 5:**
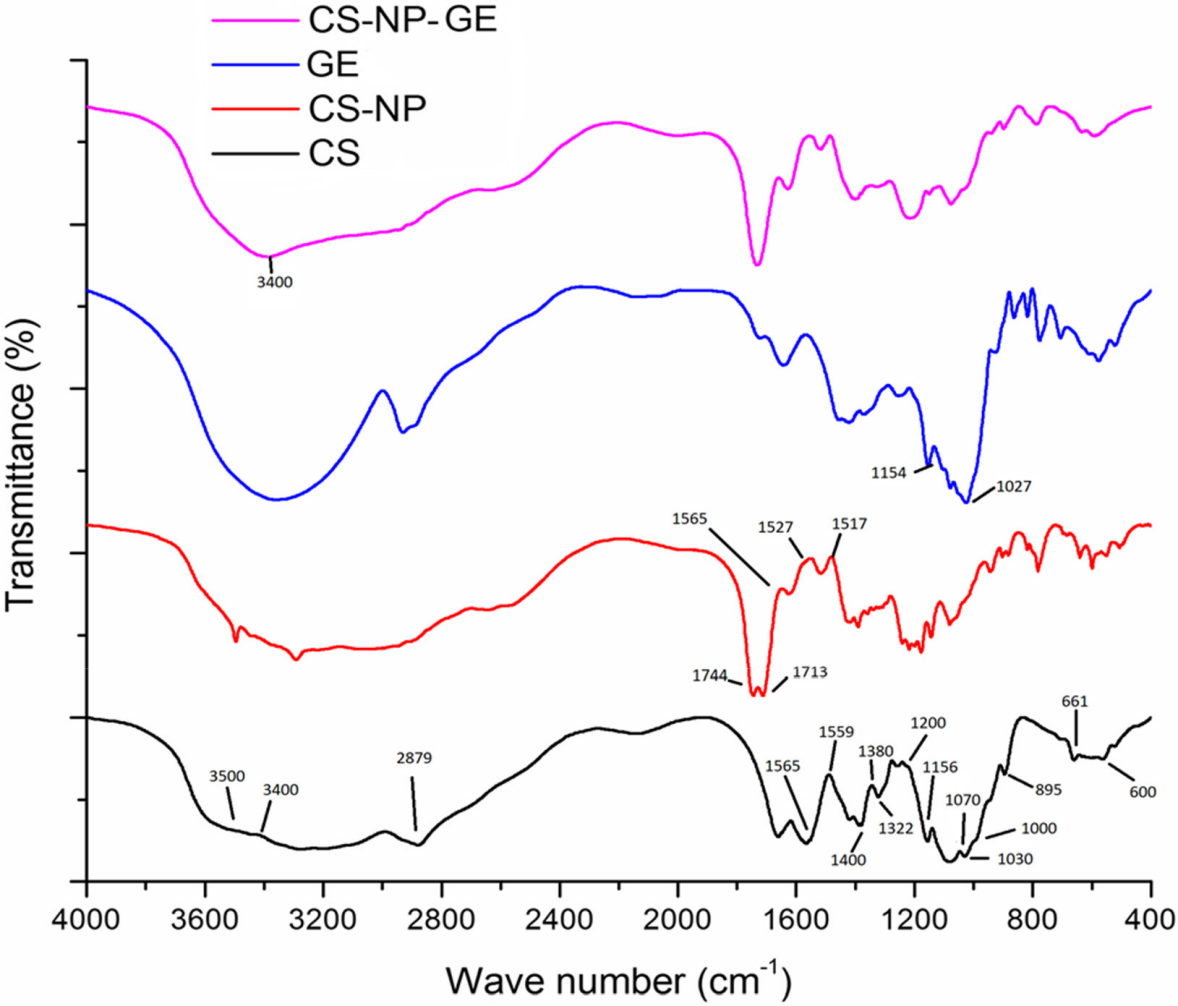
Fourier‐transform infrared (FTIR) diagram of the chitosan powder (CS), empty chitosan NPs (Empty CS‐NP), grape extract (GE), and extract‐loaded chitosan NPs (Loaded CS‐NP) at the extract/chitosan weight ratio of 1:0.5

Morphological characterization of the empty (Figure 4a) and extract‐loaded chitosan NPs (Figure 4b) was conducted utilizing the SEM technique (Figure 4). For morphological characterization, the chitosan/extract ratio of 1:0.5 was selected, as the physicochemical properties, encapsulation efficiency, and loading capacity were favorable. It can be seen in Figure 4 that the NPs are spherical with the particle size ranging between 100 and 200 nm, as confirmed by the DLS technique, and no particle accumulation can be observed. Some agglomerated particles can be seen in Figure 5, which can be due to softening process during the NP synthesis. According to Rasaee et al. (2016), *Ocimum basilicum* extract‐loaded chitosan NPs showed similar morphological properties. In the previous study of Yoksan et al. (2010), empty and ascorbyl palmitate‐loaded chitosan NPs were found to be in the range of 25–50 nm and 60–100 nm. Qi et al. (2004) reported that chitosan NP size was equal to 74.94 nm and chitosan NP with copper was equal to 47.72 nm in size. Yoksan et al. (2010) reported the chitosan NP size with and without carvacrol to be in the 40–80 nm range. Haider et al. (2017) reported the chitosan NP size to be loaded with krill oil in 100–300 nm.

### 3.6 Fourier‐ transform infrared (FTIR) spectrometry

Figure 5 indicates the FTIR spectra of the grape extract, chitosan, chitosan NPs, and extract‐loaded chitosan NPs. The spectrum of chitosan molecules displays peaks at 1565 cm^−1^ and 1659 cm^−1^, where the main tension forces between carbonyl groups (C=O) and amino groups (N–H2) are present, respectively. The symmetrical and nonsymmetrical extensions of C=O at 1659 cm^−1^ and around 1400 cm^−1^ can be attributed to the residuals and rigid chitin crust. In addition, the primary amino groups in chitosan have critical absorption between 3500 cm^−1^ and 3400 cm^−1^, which can be explained by symmetrical and nonsymmetrical tension of the N–H bond. On the other hand, there is an overlap between the strong absorption due to N–H2 vibrating tension with the ‐OH group and the formation of a latitudinal peak between 3400 and 3500 cm^−1^. A powerful configuration can be seen at 2879 cm^−1^ which can be explained by the tension vibration of C–H (allylic) groups in the chitosan circle. The absorbed peak between 1000 cm^−1^and 1200 cm^−1^ can be attributed to the saccharide structure of the chitosan skeleton. In addition, the peak at 1000–1200 cm^−1^ can be attributed to the saccharide structure of the chitosan skeleton. Absorption at 1380 cm^−1^, 1322 cm^−1^, and 1156 cm^−1^ can be attributed to C–O, O–H tension in‐plane bending, and C–O–C bonds (glucosidic bonds between chitosan bonds), respectively. Moreover, the peaks at 1070 cm^−1^ and 1030 cm^−1^ can be attributed to C–OH tension and N–H vibration, respectively. The absorption at 895 cm^−1^ is due to the vibrational tension of C–C. The peak at 661 cm^−1^ is an indicator of N–H, and vibration bending in the chitosan circle occurs in the region of 600 cm^−1^. Other studies have reported similar peaks (Barzegar et al., 2016; Ghaderi‐Ghahfarokhi et al., 2017; Haider et al., 2017; Hu et al., 2012; Jamil et al., 2016; Liang et al., 2011; Qi et al., 2004; Shahbazi & Shaveisi, 2018; Rasaee et al., 2016; Siripatrawan & Harte, 2010; Yoksan et al., 2010).

Chitosan chains create cross‐linking with chemical groups of electron donors throughout polymerization in crust environment (skeleton). Donors such as acetic acid can create a connection between carboxylic and amino groups of chitosan. FTIR spectrum of chitosan and empty chitosan NPs indicates that the rigid structure of chitosan forms covalent cross‐linking with acetic acid. The bonds are formed between ‐NH2 groups in chitosan and ‐COOH groups in acetic acid. The peaks at 1565 cm^−1^ and 1559 cm^−1^ changed to 1517 cm^−1^ and 1527 cm^−1^, respectively. The decrease in absorption in this range is due to the ‐NH2 and C=O vibrations in the chitosan skeleton. These observations confirm the chemical reaction (cross‐linking) between acetic acid and chitosan. Strong absorption with a double peak at 1744 cm^−1^ and 1713 cm^−1^ is due to the tension vibration of residual carbonyl groups from acetic acid, indicating polar molecule absorption in the encapsulation process. Other studies have reported similar peaks for chitosan NP (Haider et al., 2017; Jamil et al., 2016; Keawchaoon & Yoksan, 2011; Liang et al., 2011; Qi et al., 2004; Rasaee et al., 2016).

In the FTIR spectrum of grape extract, the critical absorption at 1027 cm^−1^ is due to the C–O tension of the hydroxyl saccharin group. In addition, stretched oxygen bridge at 1154 cm^−1^ indicates a saccharin structure. Other studies have reported similar peaks (Ghaderi‐Ghahfarokhi et al., 2016; Ghaderi‐Ghahfarokhi et al., 2017; Haider et al., 2017; Shahbazi & Shaveisi, 2018).

FTIR peak range and position change in extract‐loaded chitosan NP indicate that glucosidal chain length is precisely adjusted during the encapsulation process. The hydroxyl group of extract‐loaded chitosan NPs shows that the diversity and peak range are due to the formation of hydrogen links between the hydroxyl groups of chitosan with polyphenol groups of the grape extract. These findings indicate that the grape extract was successfully encapsulated in chitosan NPs. Similar results have been reported on the encapsulation of bioactive compounds in chitosan NPs, which are consistent with our findings (Haider et al., 2017; Jamil et al., 2016; Rasaee et al., 2016; Siripatrawan & Harter, 2010).

### 3.7 X‐ray diffraction (XRD) analysis

**FIGURE 6:**
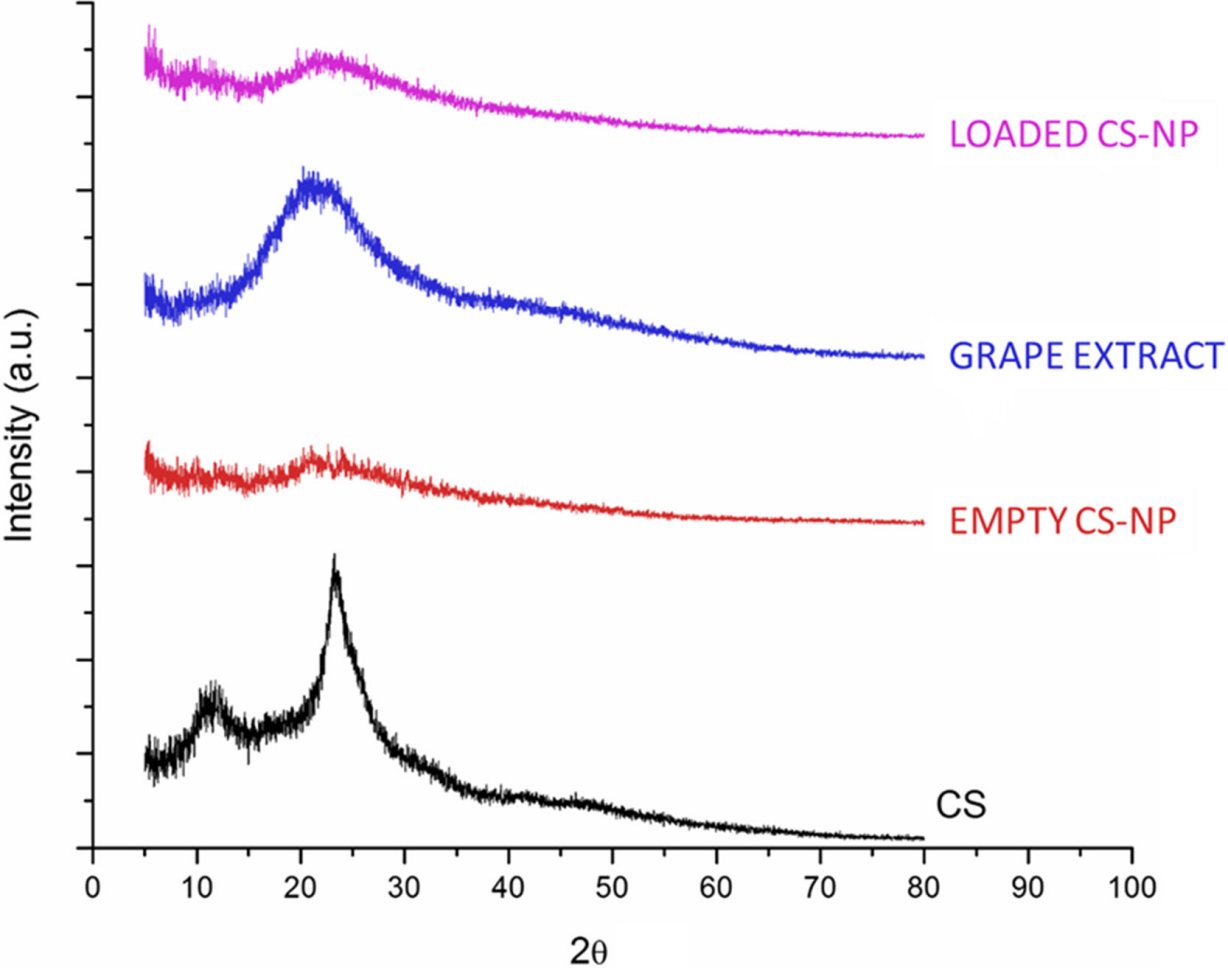
X‐ray powder diffraction technique (XRD) patterns of chitosan powder (CS), empty chitosan NPs (Empty CS‐NP), grape extract (GE), and extract‐loaded chitosan NPs (Loaded CS‐NP) at the extract/chitosan weight ratio of 1:0.5

The XRD method is used to analyze and determine the crystal structure of the chitosan NPs. The method is based on X‐ray radiation on the sample from different angles and analyzing the diffraction or reflection. The XRD patterns of the grape extract, chitosan, empty chitosan NPs, and extract‐loaded chitosan NPs are shown in Figure 6. Chitosan exhibits two reflection falls: the reflection at 2*θ* = 11.95°, which confirms the formation of crystal I, and powerful reflection at 2*θ* = 23.4°, indicating crystal II formations. Notably, chitosan is always covered with hydrated bonds (5%), even when dried out. Crystal peak at the center around (peak II) indicates the formation of crystalline structures that are formed uniformly with moderation. The peak at about 2*θ* = 34.4° indicates a high level of crystallization in chitosan powder. In the XRD pattern of chitosan NPs, the peak at 2*θ* = 11.95° is eliminated, and the peak at 2*θ* = 23.3° is extended; therefore, the strength of the last peak is lowered.

Based on these observations, the amorphous nature of chitosan NPs chains is notable, which means that cross‐linking agents (acetic acid molecules) in chitosan chains are dissolved during the formation of NPs. Therefore, creating cross‐linking prevents the reformation of chitosan chains in crystalline bulk structures. The extract demonstrated a broad peak at 2*θ* = 21.57°, which is indicative of the amorphous nature of the extract. Compared to other patterns, there is no notable difference between the XRD pattern of empty and loaded chitosan NPs. These results indicate that the amorphous nature of NPS after the encapsulation process was not eliminated, and that the loaded NPs kept their amorphous nature. In this regard, the distinctive peaks of the grape extract (2*θ* = 21.57°) and the NPs (2*θ* = 21.57°) were combined in the loading system, and only one broad peak appeared at 2*θ* = 23°. Similar results have been reported by Haider et al. (2017), Qi et al. (2004), Keawchaoon & Yoksan, (2011), and Rasaee et al. (2016).

## 4 CONCLUSION

Nanoencapsulation of the grape extract in chitosan NPs was successfully performed through the ionic gelation technique. Chitosan/extract concentration of 1:0.5 was the optimum ratio as the desired physicochemical properties, encapsulation efficiency, and loading capacity were obtained. Chitosan NPs loaded with the extract had a higher antioxidant activity compared to the free extract. The highest antioxidant activity was observed with the chitosan/extract ratio of 1:0.5. Although encapsulation of reactive agents within chitosan NPs was challenged with issues such as instability and insolubility, the findings indicated that the encapsulated grape extract with chitosan NPs had a higher antioxidant property and, in turn, could provide a higher shelf‐life and stability to the various food products.

## References

[fsn32891-bib-0001] Alishahi, A. , Mirvaghefi, A. , Tehrani, M. R. , Farahmand, H. , Shojaosadati, S. A. , Dorkoosh, F. A. , & Elsabee, M. Z. (2011). Shelf life and delivery enhancement of vitamin C using chitosan nanoparticles. Food Chemistry, 126(3), 935–940.

[fsn32891-bib-0002] Alves, V. L. , Rico, B. P. , Cruz, R. M. , Vicente, A. A. , Khmelinskii, I. , & Vieira, M. C. (2018). Preparation and characterization of a chitosan film with grape seed extract‐carvacrol microcapsules and its effect on the shelf‐life of refrigerated Salmon (Salmo salar). Lwt ‐ Food Science and Technology, 89, 525–534.

[fsn32891-bib-0009] Barzegar, M. , Ghaderi‐Ghahfarokhi, M. , Sahari, M. A. , & Azizi, M. H. (2016). Enhancement of thermal stability and antioxidant activity of thyme essential oil by encapsulation in chitosan nanoparticles. Journal of Agricultural Science and Technology, 18(7), 1781–1792.

[fsn32891-bib-0003] Cai, Y. , & Lapitsky, Y. (2017). Analysis of chitosan/tripolyphosphate micro‐and nanogel yields is key to understanding their protein uptake performance. Journal of colloid and interface science, 494, 242–254. 28160708 10.1016/j.jcis.2017.01.066

[fsn32891-bib-0004] Čalija, B. , Cekić, N. , & Milić, J. (2017). Influence of Polycation functional properties on Polyanion micro/nanoparticles for NSAIDs reinforced via polyelectrolyte complexation: Alginate–chitosan case study. In Microsized and nanosized carriers for nonsteroidal anti‐inflammatory drugs (pp. 133–160). Academic Press.

[fsn32891-bib-0005] Chandrasekaran, M. , Kim, K. D. , & Chun, S. C. (2020). Antibacterial activity of chitosan nanoparticles: A review. Processes, 8(9), 1173.

[fsn32891-bib-0006] Cho, E. J. , Holback, H. , Liu, K. C. , Abouelmagd, S. A. , Park, J. , & Yeo, Y. (2013). Nanoparticle characterization: state of the art, challenges, and emerging technologies. Molecular pharmaceutics, 10(6), 2093–2110. 23461379 10.1021/mp300697h PMC3672343

[fsn32891-bib-0007] Dube, A. , Ng, K. , Nicolazzo, J. A. , & Larson, I. (2010). Effective use of reducing agents and nanoparticle encapsulation in stabilizing catechins in alkaline solution. Food Chemistry, 122(3), 662–667.

[fsn32891-bib-0008] Ghaderi‐Ghahfarokhi, M. , Barzegar, M. , Sahari, M. A. , Gavlighi, H. A. , & Gardini, F. (2017). Chitosan‐cinnamon essential oil nano‐formulation: Application as a novel additive for controlled release and shelf life extension of beef patties. International journal of biological macromolecules, 102, 19–28. 28380334 10.1016/j.ijbiomac.2017.04.002

[fsn32891-bib-0010] Gibis, M. , Rahn, N. , & Weiss, J. (2013). Physical and oxidative stability of uncoated and chitosan‐coated liposomes containing grape seed extract. Pharmaceutics, 5(3), 421–433. 24300515 10.3390/pharmaceutics5030421 PMC3836620

[fsn32891-bib-0011] Ha, H. K. , Kim, J. W. , Lee, M. R. , & Lee, W. J. (2013). Formation and characterization of quercetin‐loaded chitosan oligosaccharide/β‐lactoglobulin nanoparticle. Food research international, 52(1), 82–90.

[fsn32891-bib-0012] Haider, J. , Majeed, H. , Williams, P. A. , Safdar, W. , & Zhong, F. (2017). Formation of chitosan nanoparticles to encapsulate krill oil (Euphausia superba) for application as a dietary supplement. Food Hydrocolloids, 63, 27–34.

[fsn32891-bib-0013] Hu, B. , Ting, Y. , Zeng, X. , & Huang, Q. (2012). Cellular uptake and cytotoxicity of chitosan–caseinophosphopeptides nanocomplexes loaded with epigallocatechin gallate. Carbohydrate Polymers, 89(2), 362–370. 24750731 10.1016/j.carbpol.2012.03.015

[fsn32891-bib-0014] Jamil, B. , Habib, H. , Abbasi, S. , Nasir, H. , Rahman, A. , Rehman, A. , Bokhari, H. , & Imran, M. (2016). Cefazolin loaded chitosan nanoparticles to cure multi drug resistant Gram‐negative pathogens. Carbohydrate polymers, 136, 682–691. 26572401 10.1016/j.carbpol.2015.09.078

[fsn32891-bib-0015] Keawchaoon, L. , & Yoksan, R. (2011). Preparation, characterization and in vitro release study of carvacrol‐loaded chitosan nanoparticles. Colloids and surfaces B: Biointerfaces, 84(1), 163–171. 21296562 10.1016/j.colsurfb.2010.12.031

[fsn32891-bib-0016] Koponen, J. M. , Happonen, A. M. , Mattila, P. H. , & Törrönen, A. R. (2007). Contents of anthocyanins and ellagitannins in selected foods consumed in Finland. Journal of agricultural and food chemistry, 55(4), 1612–1619. 17261015 10.1021/jf062897a

[fsn32891-bib-0017] Kumari, A. , Yadav, S. K. , Pakade, Y. B. , Singh, B. , & Yadav, S. C. (2010). Development of biodegradable nanoparticles for delivery of quercetin. Colloids and Surfaces B: Biointerfaces, 80(2), 184–192. 20598513 10.1016/j.colsurfb.2010.06.002

[fsn32891-bib-0018] Liang, J. , Li, F. , Fang, Y. , Yang, W. , An, X. , Zhao, L. , Xin, Z. , Cao, L. , & Hu, Q. (2011). Synthesis, characterization and cytotoxicity studies of chitosan‐coated tea polyphenols nanoparticles. Colloids and Surfaces B: Biointerfaces, 82(2), 297–301. 20888740 10.1016/j.colsurfb.2010.08.045

[fsn32891-bib-0019] Omara, N. A. , Elsebaie, E. , Kassab, H. , & Salama, A. (2019). Production of chitosan from shrimp shells by microwave technique and its use in minced beef preservation. Slov. Veter. Res., 56, 773–780. 10.26873/SVR-818-2019

[fsn32891-bib-0020] Qi, L. , Xu, Z. , Jiang, X. , Hu, C. , & Zou, X. (2004). Preparation and antibacterial activity of chitosan nanoparticles. Carbohydrate research, 339(16), 2693–2700. 15519328 10.1016/j.carres.2004.09.007

[fsn32891-bib-0021] Rajaei, A. , Barzegar, M. , Hamidi, Z. , & Sahari, M. A. (2010). Optimization of extraction conditions of phenolic compounds from pistachio (Pistachia vera) green hull through response surface method. Journal of Agricultural Science and Technology, 12, 605–615.

[fsn32891-bib-0022] Rasaee, I. , Ghannadnia, M. , & Honari, H. (2016). Antibacterial properties of biologically formed chitosan nanoparticles using aqueous leaf extract of Ocimum basilicum. Nanomedicine Journal, 3(4), 240–247.

[fsn32891-bib-0023] Roostaee, M. , Barzegar, M. , Sahari, M. A. , & Rafiee, Z. (2017). The enhancement of pistachio green hull extract functionality via nanoliposomal formulation: studying in soybean oil. Journal of food science and technology, 54(11), 3620–3629. 29051657 10.1007/s13197-017-2822-2 PMC5629171

[fsn32891-bib-0024] Shah, B. R. , Zhang, C. , Li, Y. , & Li, B. (2016). Bioaccessibility and antioxidant activity of curcumin after encapsulated by nano and Pickering emulsion based on chitosan‐tripolyphosphate nanoparticles. Food Research International, 89, 399–407. 28460931 10.1016/j.foodres.2016.08.022

[fsn32891-bib-0025] Shahbazi, Y. , & Shavisi, N. (2018). Characterization of active nanochitosan film containing natural preservative agents. Nanomedicine Research Journal, 3(2), 109–116.

[fsn32891-bib-0026] Silva, V. , Singh, R. K. , Gomes, N. , Soares, B. G. , Silva, A. , Falco, V. , Capita, R. , Alonso‐Calleja, C. , Pereira, J. E. , Amaral, J. S. , Igrejas, G. , & Poeta, P. (2020). Comparative insight upon chitosan solution and chitosan nanoparticles application on the phenolic content, antioxidant and antimicrobial activities of individual grape components of Sousão variety. Antioxidants, 9(2), 178. 10.3390/antiox9020178 PMC7070837 32098120

[fsn32891-bib-0028] Siripatrawan, U. , & Harte, B. R. (2010). Physical properties and antioxidant activity of an active film from chitosan incorporated with green tea extract. Food hydrocolloids, 24(8), 770–775.

[fsn32891-bib-0029] Tang, D. W. , Yu, S. H. , Ho, Y. C. , Huang, B. Q. , Tsai, G. J. , Hsieh, Sung, H.‐W , & Mi, F. L. (2013). Characterization of tea catechins‐loaded nanoparticles prepared from chitosan and an edible polypeptide. Food Hydrocolloids, 30(1), 33–41.

[fsn32891-bib-0030] Vekiari, S. A. , Gordon, M. H. , Garcia‐Macias, P. A. , & Labrinea, H. (2008). Extraction and determination of ellagic acid contentin chestnut bark and fruit. Food Chemistry, 110(4), 1007–1011. 26047294 10.1016/j.foodchem.2008.02.005

[fsn32891-bib-0031] Yoksan, R. , Jirawutthiwongchai, J. , & Arpo, K. (2010). Encapsulation of ascorbyl palmitate in chitosan nanoparticles by oil‐in‐water emulsion and ionic gelation processes. Colloids and Surfaces B: Biointerfaces, 76(1), 292–297. 20004558 10.1016/j.colsurfb.2009.11.007

[fsn32891-bib-0033] Zarei, M. , Ramezani, Z. , Ein‐Tavasoly, S. , & Chadorbaf, M. (2015). Coating effects of orange and pomegranate peel extracts combined with chitosan nanoparticles on the quality of refrigerated silver carp fillets. Journal of food processing and preservation, 39(6), 2180–2187.

[fsn32891-bib-0032] Zhang, H. , Jung, J. , & Zhao, Y. (2016). Preparation, characterization and evaluation of antibacterial activity of catechins and catechins–Zn complex loaded β‐chitosan nanoparticles of different particle sizes. Carbohydrate Polymers, 137, 82–91. 26686108 10.1016/j.carbpol.2015.10.036

[fsn32891-bib-1032] Zorzi, G. K. , Carvalho, E. L. S. , Von Poser, G. L. , & Teixeira, H. F. (2015). Revista Brasileira de Farmacognosia. 25, 426–436.

